# Stability of and Changes in Implicit Motives. A Narrative Review of Empirical Studies

**DOI:** 10.3389/fpsyg.2018.00777

**Published:** 2018-05-25

**Authors:** Ferdinand Denzinger, Veronika Brandstätter

**Affiliations:** Department of Psychology - Psychology of Motivation, Volition, and Emotion, University of Zurich, Zurich, Switzerland

**Keywords:** implicit motives, review, change, stability, retest, age differences, arousal

## Abstract

Although growing research indicates that certain personality traits change over the lifespan, implicit motives are often deemed to be rather stable personality characteristics. Researchers have been interested in implicit motives for several decades, but our understanding of how these dispositions change still lacks clarity. This article gives an overview and a discussion of the current evidence for the stability of and the changes in implicit motives. After elaborating on the theoretical background of the motive construct and its measurement, we present an overview of studies that have investigated the trainability of implicit motives and their dispositional stability and changes using cross-sectional and longitudinal methods. Although the results are inconclusive concerning the direction of change, the reviewed studies suggest that implicit motives adapt to life circumstances much like other personality traits. This review sets out to contribute to a better understanding of the functioning of implicit motives and to present a roadmap for further research.

“When you're finished changing, you're finished”

-Benjamin Franklin

The above quote reveals that our entire life is characterized by different and inevitable changes, such as life transitions, critical life events, or age-related psychological and physiological changes. Researchers in the domain of personality psychology have acknowledged that such changes also affect personality characteristics. They conclude that personality traits (e.g., the big five) are not as stable as has long been assumed. Recent studies have shown that they change over the course of one's life (for an overview, see Roberts et al., 2006; Specht et al., 2011). A meta-analysis based on 92 studies on changes in personality traits showed significant changes in various personality dispositions, for example, a decrease in openness and an increase in conscientiousness, over the course of one's life (Roberts et al., 2006). However, such changes do not only affect the big five, but also other personality characteristics such as self-confidence, self-control, and intelligence (e.g., Verhaeghen and Salthouse, 1997; Roberts and Mroczek, 2008). In sum, there is empirical evidence that personality characteristics do indeed change.

Researchers have proposed various metrics for assessing and describing the stability of and changes in personality characteristics (e.g., Caspi and Roberts, 2001). Often, researchers refer to mean-level and rank-order consistency as two indices of stability and change. Mean-level consistency can also be described as normative stability. This metric “reflects shifts of groups of people to higher or lower values on a trait over time” (Specht et al., 2011, p. 863). An example might be the decrease of openness in aging adults (Roberts et al., 2006). The rank-order consistency is also called differential stability. It addresses the individual deviations from such normative trends and is, for example, typically indicated by correlation coefficients such as the retest-stability coefficient (Caspi and Roberts, 2001). Both indices are of interest and can occur independently of each other. It seems likely that such changes over the course of one's life do not only affect the above-mentioned personality traits, but also basic motivational orientations such as implicit motives. These can be considered another core part of one's personality (Winter, 1996) that covers a different aspect of it and also predicts different behavior than traits such as the big five (Winter et al., 1998). Implicit motives are defined as unconscious motivational dispositions that are activated through affectively charged incentives influencing spontaneous behavior (McClelland, 1985; Schultheiss, 2008). Researchers postulate that they are acquired early in the preverbal stage of life, through the repeated experience of motive-satisfying incentives (McClelland and Pilon, 1983). Despite the growing understanding about changes in personality characteristics (e.g., Roberts et al., 2006), implicit motives are often still considered to be a comparatively stable part of an individual's personality that is exclusively formed in the early childhood (McClelland and Pilon, 1983; McClelland, 1985). Although research on implicit motives goes back more than 60 years, the stability assumption of implicit motives has rarely been questioned and there has been only little research on this issue. The purpose of this article is to offer an overview of existing empirical evidence and the related theoretical assumptions about the stability of and changes in implicit motives. Research on implicit motives is currently enjoying a revival (e.g., Schultheiss and Brunstein, 2010). Thus, it is of paramount importance to get a general idea of different propositions and theoretical points of view concerning the stability of and changes in implicit motives, since this will open up new avenues for future research.

## Implicit motives and their origins

### Implicit motives: theoretical background, definition, and measurement

Implicit motives are enduring preferences for “the attainment of specific classes of incentives and the avoidance of specific classes of disincentives” (Schultheiss, 2008, p. 603). Thus, they influence the perception and evaluation of specific situations (Heckhausen, 1977). These motivational dispositions individually orient and energize one's behavior toward situations that have already been experienced as or are at least anticipated as being rewarding (McClelland, 1985). For example, some individuals might experience positive emotions in a group discussion when influencing others, while other individuals might be happy in the same situation through making new friends.

Implicit motives are not readily accessible to the conscious mind and, therefore, not assessable using self-reports of personal needs (McClelland et al., 1989). Instead, they are primarily assessed using indirect measures which rely on projective techniques that instruct individuals to produce imaginative stories based on ambiguous picture stimuli that depict people in different situations (e.g., a couple sitting on a bench by the river or a ship's captain talking to another man; Pang, 2010). Projective techniques for the measurement of implicit motives are based on the underlying assumption that ambiguous picture cues arouse specific needs. These influence the content of the individual's fantasy and are projected onto the characters of the stories which the individual writes about these pictures (McClelland, 1985; Pang, 2010). Consequently, this motivational response emerges through the contents of the written imaginative material and can be coded for its motive imagery using standardized and validated content coding systems (e.g., McAdams, 1980; Winter, 1991). The most frequently used measure of implicit motives is the Picture Story Exercise (PSE, McClelland et al., 1989; Pang, 2010) which is based on the Thematic Apperception Test (TAT, Morgan and Murray, 1935). However, researchers have also developed other motive measures such as the semi-projective Multi-Motive Grid (MMG; Sokolowski et al., 2000), the Operant Motive Test (OMT; Kuhl et al., 2003), domain-specific approaches such as the Partner-Related Agency and Communion Test (PACT; Hagemeyer and Neyer, 2012), several measures based on the Implicit Associations Test (IAT; Brunstein and Schmitt, 2004; Slabbinck et al., 2011), and physiology-based measures (Dufner et al., 2015). These tests differ, for example, in their response format (e.g., open, structured, and semistructured format) and regarding the way the motive content is coded (e.g., the OMT additionally assesses implementation strategies for satisfying implicit motives).

### The “big three”

Since the 1950s, researchers have been interested in the “big three” of implicit motives. McClelland (1985) called them the need for Achievement (*n* Ach), the need for Power (*n* Pow) and the need for Affiliation (*n* Aff). The achievement motive is the need to accomplish something difficult in competition with a high and challenging standard of excellence. The power motive is the need for the experience of having an impact on others. The affiliation motive is the need to establish and maintain positive relationships with others (McClelland, 1985; Schultheiss, 2008). Implicit motives in these three different domains represent a capacity to derive satisfaction from the attainment of the above-mentioned domain-specific incentives (e.g., mastering a challenge; Schultheiss, 2008). A multitude of studies have demonstrated that implicit motives are a good predictor of individual behavior in all motive domains and, subsequently, highlighted the importance of implicit motives for an individual's life (e.g., DeCharms et al., 1955; McClelland, 1961, 1965, 1987; McClelland and Boyatzis, 1982; McAdams and Constantian, 1983; Biernat, 1989; Jenkins, 1994; Brunstein and Hoyer, 2002; Schultheiss and Brunstein, 2002; Hagemeyer et al., 2016).

However, implicit motives are not only related to the individual's behavior, but also to psychophysiological responses such as the release of hormones (implicit power motive: testosterone and estradiol; implicit achievement motive: cortisol and vasopressin; implicit affiliation motive: dopamine and progesterone; McClelland et al., 1987; Schultheiss and Rohde, 2002; Schultheiss, 2013; Schultheiss et al., 2014; Yang et al., 2015). In addition, implicit motives are associated with specific health outcomes such as stress, changes in blood pressure and immune parameters (McClelland, 1989; Baumann et al., 2005; Schüler et al., 2009; Job et al., 2010; Brandstätter et al., 2016). In sum, implicit motives are an integral part of the human being. They influence individual behavior and are strongly linked to various psychological and physiological processes.

### Short-term changes in motivational states as a precondition for dispositional change

As mentioned above, the individual implicit motive score is derived from imaginative stories written by individuals in response to specific picture cues. McClelland et al. (1953) assumed that the contents of individuals' fantasies represent their actual state of motivation resulting from the interaction of the person's motive disposition (e.g., implicit achievement motive) and situational motive-relevant cues (e.g., situational cues interpretable in terms of challenge and excellence; c.f. the distinction between motive and motivation: Rheinberg, 2008). On the basis of this motive-motivation principle, McClelland et al. (1953) developed an implicit motive coding system. They chose an experimental approach to derive the coding categories of the particular implicit motives: for the achievement motive, for example, one group of participants was asked to write their imaginative stories under neutral testing conditions (no situational cues relevant to the motive), while the motivational states of the other group of participants were aroused beforehand (e.g., through simulating a testing situation). Consequently, the latter stories were full of content that matches the particular implicit motive. Afterwards, the stories of the two conditions were compared and the emerging content associated with the motive was used to form coding categories for that particular implicit motive-based on the assumption that the stories were written under neutral conditions. McClelland (1985) described the contents of the imaginative stories that thematically match the motive as the “unique effect of the motive and […] as an index of the strength of the motive” (p. 185). Consequently, if individuals write stories that are full of content that matches a specific motive without being motivationally aroused beforehand, individuals seem to score very highly with regard to this specific implicit motive (Schultheiss and Brunstein, 2010).

In summary, the sensitivity to the experimental arousal was an important criterion for implicit motive measures (McClelland, 1958, 1985; Winter, 1998). Schultheiss and Brunstein (2010) also described this as a common principle of implicit motive research. In consequence of this experimental derivation of the measurement and the coding categories, the TAT/PSE meets the sensitivity criterion of a valid motive measure (McClelland, 1985) as the arousal of a motive ought to increase the respective motive score. This situational susceptibility highlights the character of implicit motivation as a state. As used by personality theorists, the term “state” corresponds to an aroused motive while the term “trait” corresponds to the underlying motive disposition (McClelland, 1985).

Up to now, a vast body of research exists on the experimental arousal of implicit motives. Basically, two different kinds of studies can be distinguished: (a) studies for validation purposes, using methods that ought theoretically to arouse a specific motivational state (e.g., McClelland et al., 1953; Veroff, 1957; Uleman, 1972) and (b) studies for evaluating the motivational meaning of specific situations, substances and treatments. Many researchers have aroused implicit motives through the confrontation with motive-specific cues, such as visualizations, imagination tasks or situationally prompted motive-relevant behavior. Overall, they have demonstrated that these environmental cues influence implicit motive scores. For example, researchers presented motive-arousing movie excerpts lasting between about half an hour and one hour to individuals (e.g., an excerpt of the movie “The Godfather II” to arouse implicit power motives; Schultheiss et al., 2004; or an excerpt of a movie about the activities of Mother Teresa to arouse implicit affiliation motives; McClelland and Kirshnit, 1988) and documented significant increases in the respective implicit motive scores after individuals had watched these movie excerpts (McClelland and Kirshnit, 1988; Schultheiss et al., 2004; Wirth and Schultheiss, 2006). In addition, a recent study investigated the effects of a guided visualization designed to arouse the achievement motive on the implicit achievement motive (Rawolle et al., 2016). In this setting, participants had to close their eyes and listen to the vivid description of an achievement-related scenario (e.g., about a graduation ceremony). The authors reported higher achievement motive scores after arousal, too (Rawolle et al., 2016).

Some researchers have also used specific behavioral tasks to arouse implicit motives. Haber and Alpert (1958) employed specific achievement-oriented instructions for the TAT assessment of the implicit achievement motive (e.g., they instructed participants to do their best and emphasized a testing situation), as well as carrying out achievement tests beforehand to arouse the implicit achievement motive. They found a significant increase in achievement imagery from measurements before and after the behavioral achievement task. Another group of researchers used a stress test that reliably increases the cortisol concentration, and thus the (power-related) psychosocial stress, to arouse implicit motives (Wiemers et al., 2015). They instructed their participants to give a talk in front of a panel of judges, who were instructed to maintain neutral expressions during the talk, and additionally videotaped them. They found that this power-related stress test increased the implicit power motive but not the implicit achievement and affiliation motive scores (Wiemers et al., 2015). McClelland et al. (1972) found similar results when investigating the effects of alcohol consumption on the implicit power motive. They invited men to specific natural social settings (e.g., a party, a local bar) and assessed their implicit motives before and after ordering and consuming alcoholic beverages. The researchers found that drinking tends to increase generalized power concerns and, in turn, the implicit power motive scores.

The authors' arguments about the situational susceptibility of implicit motives are mostly based on fundamental theoretical assumptions about the operation of implicit motives that are concisely summarized by a chapter in a book by Schultheiss (2008). Among other things, these assumptions imply that persons are sensitive to specific motive-relevant cues. These cues are able to arouse an implicit motive because they indicate possible affective rewards, for example, and thus energize behavior to secure the rewards. As mentioned above, it is assumed that exposure to motive-specific cues fosters motive-specific content in fantasies (e.g., Wirth and Schultheiss, 2006; Wiemers et al., 2015; Rawolle et al., 2016). These picture-like mental representations of motive-relevant situations in individuals' stream of thoughts facilitate access to the emotional and motivational systems associated with implicit motives and might be able to engage these systems, inducing aroused motivational states (Schultheiss et al., 2004; Wirth and Schultheiss, 2006). McClelland and his colleagues argue that the confrontation with motive-specific cues also results in a physiological arousal, which influences the motivational states (McClelland et al., 1972; McClelland and Kirshnit, 1988). For example, they assumed that alcohol consumption stimulates the sympathetic nervous system (e.g., release of adrenalin, increased alertness, increased pulse rate, dulled pain), which might cause increased thoughts about power and strength and “make a man feel stronger” (McClelland et al., 1972, p. 282). Additionally, individuals with a high implicit power motive become emotionally aroused by power-related movies (e.g., they get angry) and enter a state of sympathetic arousal. This, in turn, influences their fantasy production increasing the implicit power motive score. In the same vein, an affiliation-arousing movie leads to more relaxation and positive feelings in people with an affiliative orientation, which, in turn, increases the implicit affiliation motive score.

To summarize, researchers have been able to arouse implicit motives by confronting subjects with motive-specific cues (e.g., imagination tasks, preceding behavior). They argued that changes in the implicit motive scores are due to the arousal of the motivational system. This arousal can take place on the physiological level, through specific physiological changes (e.g., release of hormones), as well as on the psychological level, through facilitated access to the motivational and emotional system after the perception of motive-relevant cues. Both effects result in an improved accessibility and strength of the associative implicit motive network. The above-mentioned studies give evidence that specific environmental cues influence the motivational state. Presumably, the experimental arousal and the short-term change of motivational states are important for long-term changes in the implicit motive disposition, because this basic situational susceptibility of implicit motives might be a precondition for long-term influences on implicit motives. It seems important to address this issue at the beginning of this review, since long-term changes in implicit motive dispositions might come about through repeated changes in motivational states.

### Origins and development of implicit motives

Implicit motives exist in all human beings, but are expressed to different degrees in each individual. Researchers conclude that the capacity to develop implicit motives is innate and that individual differences are mainly attributable to early (prelinguistic) learning experiences (Weinberger and McClelland, 1990; for recent reviews on the interaction between implicit motives and learning/memory see Schultheiss and Köllner, 2014; Schultheiss and Schultheiss, 2014). McClelland (1985) argued that implicit motives are automatic impulses triggered by environmental variables (e.g., a baby being touched by its mother) to act in a specific way (e.g., seeking contact) that enhances the likelihood of pleasurable affective experiences (e.g., feeling accepted). He termed these experiences the “natural incentives” (McClelland, 1985, p. 136).

The presence and the experience of such natural incentives may serve as a precondition to the above-mentioned early learning processes of implicit motives. McClelland's (1985) model assumes that individual differences in implicit motives emerge in line with early learning processes similar to operant conditioning. The occurrence of specific behaviors in response to specific (natural) incentives is reinforced or reduced by the positive or negative consequences of the respective behavior. For example, if an individual responds to specific cues in the environment (e.g., a challenge) and experiences positive consequences of this behavior (e.g., reward by the parents for overcoming the challenge), the individual's pleasurable feelings will increase. Subsequently, the individual will afterwards behave in a similar manner to re-experience the positive affect. This behavior and the striving for these specific satisfying incentives will be consolidated through a frequent repetition of the above-mentioned process (McClelland, 1985). To date, it has not been finally established at which developmental stage these learning processes occur and at what age they stop working. Some researchers argue that these processes already take place from the early preverbal neonatal phase (McClelland, 1985), while others argue that developmental processes happen from the age of one (Veroff, 1959), or even from the age of three (Heckhausen, 1967).

Researchers assume that these learning processes result from specific parental child-rearing practices (McClelland and Pilon, 1983; McClelland et al., 1989). First empirical evidence was furnished by a few studies that investigated the relationship between specific parenting practices in childhood and implicit motive dispositions in adulthood (e.g., Winterbottom, 1958; Rosen and D'Andrade, 1959; Rosen, 1962; McClelland and Pilon, 1983). For example, McClelland and Pilon (1983) did a follow-up study on children whose mothers were interviewed on their child-rearing practices when their children were five years old. The researchers contacted these children more than 25 years later and assessed their implicit motives. They found that strict toilet training and scheduled feeding in childhood was linked to a high implicit achievement motive. Presumably, this is caused by a strong focus on mastery and independence in parental child rearing practices. They also found that maternal permissiveness about aggressive and sexual impulses in the first years of life was linked to a high implicit power motive. Data regarding the implicit affiliation motive was less clear. Children who experienced insecurity in their early affiliative relationships (e.g., mothers not reacting to their child crying) seemed to developed a strong fear of rejection, which is a facet of a high implicit affiliation motive (McClelland and Pilon, 1983). To sum up, specific child-rearing practices might allow children to experience specific natural incentives (e.g., mastering a challenge or influencing others) as being rewarding. The repeated execution of behaviors to gain specific rewards reinforces the respective motive disposition.

Researchers argue that such affective learning experiences consolidate a strong and rather stable motive disposition exclusively in early childhood (McClelland et al., 1989) and that specific experiences in later life do not necessarily influence the implicit motive disposition (Weinberger and McClelland, 1990). However, this is basically a theoretical assumption. The learning process of implicit motives, which is comparable to an operant conditioning approach, could also occur at school-age and even in adulthood. It is theoretically possible that implicit motives are acquired and even changed later in life through comparable affective experiences and learning processes (McClelland, 1958; McClelland and Pilon, 1983; McClelland et al., 1989; Duncan and Peterson, 2010). It is conceivable that environmental influences, such as specific changes in an individual's life (e.g., increased confrontation with new situational incentives, transitions, critical life events) trigger affective learning experiences. Furthermore, recent research has even shown that implicit motives may have roots not only in prenatal (Schultheiss and Zimni, 2015) but also in the pubertal organizing effects of gonadal steroid hormones (Janson et al., 2018). Researchers in the field of personality psychology have already revealed the important role of the environment (in addition to maturation according to temperament or genetic factors) as a major underlying cause of change in most personality characteristics (e.g., Kogan, 1990; Roberts et al., 2006; Specht et al., 2011; Hudson and Fraley, 2015; Anusic and Schimmack, 2016). They assumed, for example, that individual experiences in (new) social roles (e.g., parenthood) and associated role expectations by others influence the individual personality disposition through processes similar to the above-mentioned operant conditioning (e.g., through repeated punishment, reward, or social reinforcement of expected behavioral and affective patterns; Roberts et al., 2006; Specht et al., 2011; Hudson and Fraley, 2015). However, not only expectations about social roles are deemed to influence personality characteristics, but also normative changes and major life events (Specht et al., 2011). In general, “changing circumstances can contribute to changes in personality” (Anusic and Schimmack, 2016, p. 774).

Hence, environmental influences should also be considered an important factor that might be responsible for changes in the individual implicit motive disposition. We will initially give a short overview of studies that examined intervention programs on the trainability of these personality characteristics. In a second step, we will describe studies that investigated the dispositional stability of and changes in implicit motives in the “real world” over the course of one's life. This includes studies that analyzed the influence of environmental factors such as specific life circumstances (e.g., transitions) on implicit motive scores over the course of life, using a cross-sectional as well as a longitudinal approach, with measures repeated at intervals of several weeks, months, years or even decades.

A more detailed examination of the literature on this topic reveals heterogeneous theoretical views on and explanatory approaches to change-related processes of implicit motives. Unfortunately, a large part of this research is already quite outdated and many studies are hampered by methodological problems (e.g., small sample sizes). In view of this theoretical heterogeneity and the methodological shortcomings, it would not be viable to summarize and compare these studies statistically (e.g., comparing effect sizes). However, research on implicit motives is currently witnessing a revival (e.g., Schultheiss and Brunstein, 2010). Thus, it is not only useful but also urgently necessary to take stock of previous research on the stability of and changes in implicit motives. The purpose of this article is to summarize the empirical evidence on this issue while also providing an overview of current theoretical assumptions about the underlying processes. Furthermore, a roadmap for future research will be deduced from the limitations of previous studies.

This review has great theoretical as well as practical relevance: It describes fundamental processes regarding the functioning of implicit motives and gives an overview of preconditions regarding the activation and development of implicit motives. The understanding gained from this review may be helpful in designing motivation trainings or even interventions for stress reduction, for example by reducing motive-goal incongruences.

## Method

The literature search was conducted using the databases PsychINFO, PsychARTICLES, PsychBOOKS, PSYNDEX, and the search engine Google Scholar using various combinations of the following keywords: implicit motives, change, stability, retest, training, age differences, reliability, PSE, TAT, OMG, MMG, PACT, IAT, development, life span, and longitudinal. Neither the publication dates, nor the geographical location of the studies were restricted. Furthermore, we used the ancestry approach and screened the references of all located articles for additional studies. To answer our research question, we only included those studies that conformed to the following criteria: First, implicit motives (achievement, power, affiliation) must be assessed using fantasy-based measures, namely the Thematic Apperception Test (TAT) and the Picture Story Exercise (PSE). These measures are properly validated for measuring implicit motives in the McClelland-Atkinson tradition (McClelland et al., 1989; Smith, 1992a; Schultheiss, 2008). However, as already mentioned, researchers have recently developed new and highly promising approaches to measure implicit motives. For the sake of completeness and to account for these recent developments, we will treat studies using such approaches separately at the end of the results section. Second, implicit motives must be analyzed using common and standardized coding rules [e.g., Winter's (1994) Manual for scoring motive imagery in running text]. Third, studies must be published in scientific journals or books. Fourth, studies must match the following types of studies: (a) studies that investigate changes in implicit motives that are due to implicit motive training and that assess implicit motive scores in a pre- and post-condition. Studies that (b) analyze stability and change of implicit motives with cross-sectional designs or (c) with longitudinal designs, over the course of several weeks, months, or years. In total, we selected 33 studies that comply with our criteria for this review. Further, we selected six additional studies that use alternative measure. In the following section, we have summarized these studies according to the above-mentioned procedure.

## Results

This review of literature is structured as follows: to begin with, we will focus briefly on studies that investigated the possibility of training implicit motives. Then, we give an overview of studies that have analyzed the stability of and changes in implicit motives by cross-sectional and by longitudinal methods. In each part, we briefly describe the respective studies and summarize the results, then we report the authors' theoretical assumptions about the stability of and changes in implicit motives (e.g., how do they explain the result of a change in the implicit motive pattern?).

### Studies that investigate the possibility of training implicit motives

Table 1 gives an overview of studies that investigated the possibility of training implicit motives. The training program developed by McClelland and Winter (1969) was presumably the first explicit attempt to change individuals' implicit motive disposition in the long run. The studies on motivation training investigated effects of specific exercises (e.g., theoretical inputs, self-awareness exercises) on implicit achievement and power motives in the contexts of economic growth (McClelland and Winter, 1969) and motivation in the classroom (deCharms, 1976a). Whereas, McClelland and Winter (1969) described an overall increase in implicit achievement and power motives as well as increased economic activity 2 years after the training, deCharms (1976a) reported an increase in implicit achievement motives only for boys who received the training in sixth grade, but not for girls, and neither for boys nor girls who received the training in seventh grade. The authors concluded that there are two reasons for these results: The measurement of the girls' achievement motivation was not reliable because the variability of their data appeared to be reduced. Besides, the training in the seventh grade did not stress achievement motivation. However, the authors acknowledged that their findings for training effects were not very strong.

**Table 1:** Studies investigating the trainability of implicit motives.

**Author(s)**	**Sample**	**Motive**	**Type of training**	**Change in motive(s)**
deCharms, 1976a	*N* = 88	Achievement	Achievement training in the classroom	Increase in *n* Ach only for boys in the sixth grade
McClelland and Winter, 1969	*N* = 49	Achievement, Power	Achievement training	Increase in *n* Ach and *n* Pow after training

*n Ach, implicit achievement motive; n Pow, implicit power motive*.

Researchers who investigated the effects of implicit motive trainings assumed that implicit motives can be changed to a certain degree. They considered implicit motives to be affectively toned associative networks that are shaped and stabilized in childhood as a basic part of the personality (McClelland and Winter, 1969). They concluded that environmental influences and intensive learning experiences (e.g., through a specific training) repeatedly activate these networks and can thus increase the accessibility and strength of implicit motives. DeCharms (1976b) developed his achievement motivation training on the basis of his theory of personal causation with the aim of empowering people to control their own behavior (to become “Origins”). He assumed that the concept of achievement motives is very similar to his notion of an Origin that might be changed through specific training.

### Cross-sectional analyses

The first part showed that implicit motives are personality characteristics that are susceptible to the influence of environmental factors. We will now focus on studies that were especially interested in the mean-level stability of implicit motives. The cross-sectional studies reported in this section investigated the implicit motive scores of individuals across different age groups. Although these studies did not analyze implicit motive scores with repeated measurements over a period of decades, they are highly relevant because some of the authors made sophisticated assumptions about changes in implicit motives.

Table 2 gives an overview of studies that investigated the effects of age on implicit motive scores. First, we will briefly describe the studies and summarize their results; then we will report the authors' assumptions about and interpretations of age differences in implicit motives.

**Table 2:** Cross-sectional studies investigating implicit motive scores.

**Author(s)**	**Sample**	**Age range**	**Motive(s)**	**Main results**	**Reason for change**
Denzinger et al., 2016	*N* = 735	20–35 years old, 40–55 years old, 65–80 years old	Power, Affiliation, Achievement	Lower scores of all motives in aged adults	Age-dependent changes in affective and neuro-endocrinological reactivity
McClelland et al., 1998	*N* = 153; community sample	25–40 years old (*n* = 77), 65–87 years old (*n* = 76)	Power, Affiliation, Achievement	Lower scores of *n* Aff and *n* Pow in aged adults	Changes in work and family patterns
Pang and Schultheiss, 2005	*N* = 322 students	18–35 years old	Power, Affiliation, Achievement	Negative correlation between *n* Ach and age (*r* = −0.12)	No reason described
Salili, 1996	*N* = 764 college students	13–55 years old	Achievement	Lower scores of *n* Ach in aged adults	Aged individuals have already passed achievement relevant stages (change in life circumstances)
Schultheiss and Brunstein, 2001	*N* = 428 college students	18–36 years old	Power, Affiliation, Achievement	Negative correlation between motive scores and age (*n* Pow: *r* = −0.11; *n* Aff: *r* = −0.12)	No reason described
Thielgen et al., 2015	*N* = 201	20–66 years old	Power, Affiliation, Achievement	No significant correlation between motive scores and age	Decreasing future time perspective increases perceived importance of social relations
Valero et al., 2014	*N* = 108; community sample	18–32 years old (*n* = 53), 54–86 years old (*n* = 55)	Power, Affiliation, Achievement	Higher scores of n Ach (*d* = 0.60) and *n* Aff (*d* = 0.33) in aged adults	The urgency to satisfy implicit motives is increased, when the future time perspective is perceived as limited
Veroff et al., 1960	*N* = 1619	21–34; 35–54; 55+	Power, Affiliation, Achievement	Lower scores of *n* Ach in aged adults; lower scores of *n* Aff in aged women; higher scores of hope for power for men at midlife	Generation differences and different stages of the individual life cycle
Veroff et al., 1980	1957: *N* = 1369; 1976: *N* = 1040	21–34; 35–54; 55+	Power, Affiliation, Achievement	Lower scores of *n* Aff in aged women; higher scores of hope for power for men at midlife	Large-scale social changes
Veroff et al., 1984	1957: *N* = 1363; 1976: *N* = 1208	21–34; 35–54; 55+	Power, Affiliation, Achievement	Lower scores of *n* Ach and *n* Aff in aged women; higher scores of hope for power for men at midlife	Changes in work and family patterns

*n Ach, implicit achievement motive; n Pow, implicit power motive; n Aff, implicit affiliation motive*.

Denzinger et al. (2016) carried out a cross-sectional comparison of the implicit motive scores of *N* = 735 individuals aged from 20 to 80 years and found lower scores for the implicit power, achievement, and affiliation motives in older adults compared with younger adults. Another group of researchers investigated implicit motives in two samples of two different birth cohorts, which were representative of the American population, and documented significant age differences in the implicit power, achievement and affiliation motives, too (Veroff et al., 1960, 1980, 1984). They found reduced scores for the implicit achievement motive in aging adults, lower scores for the implicit affiliation motive in aged women and a high hope of power in men at midlife which also declines in older age. Salili (1996) reported a negative relationship between age and the implicit achievement motive in a study of 764 Chinese students aged 13 to 55 years. In a study comparing the implicit motive scores of two age groups (young adults, *N* = 77, 25–40 years old; older adults, *N* = 76, 65–87 years old), other researchers reported lower scores for the implicit power and affiliation motives in older adults compared with younger adults, but not in the implicit achievement motive (McClelland et al., 1998). Schultheiss and Brunstein (2001) were not primarily interested in age differences in implicit motives in their study, but they nevertheless investigated the correlation between age and the implicit motive scores of 428 college students aged between 18 years and 36 years. They found a negative correlation between age and the implicit power and affiliation motive, but not between age and the implicit achievement motive. However, a study by Pang and Schultheiss (2005) found only a negative relationship between the implicit achievement motive and age in a sample of 322 students aged from 18 years to 35 years. Another study only found a trend toward a negative relationship between age and implicit power, achievement and affiliation motives in *N* = 201 individuals aged from 20 years to 66 years, but no significant effects (Thielgen et al., 2015). A further study even reported a positive relationship between age and implicit achievement and affiliation motives, but not between age and the implicit power motive in *N* = 108 individuals ranging from the age of 18 years to the age of 86 years (Valero et al., 2014).

To summarize, the cross-sectional results on stability and changes in implicit motives appear to be mixed, but mostly provide statistical evidence for considerable mean-level changes (age differences) in implicit motives. One study only found a trend toward a negative relationship between age and implicit motive scores (Thielgen et al., 2015), and another study even reported a positive relationship between age and implicit motive scores (Valero et al., 2014). Nonetheless, the majority of studies reported a negative relationship between age and implicit motive scores (Veroff et al., 1960, 1980, 1984; Salili, 1996; McClelland et al., 1998; Schultheiss and Brunstein, 2001; Pang and Schultheiss, 2005; Denzinger et al., 2016).

How do the authors of the respective studies interpret these results? The majority of the authors assumed that implicit motives may change over the lifespan, but researchers have different theoretical views about the mechanisms and the direction of change. Some researchers assumed that implicit motives might change with increasing age in line with changes in life circumstances as well as in work and family role commitments (Veroff et al., 1960, 1980, 1984; Salili, 1996). These factors might influence implicit motive scores in addition to generational differences and cohort effects (Veroff et al., 1960). It is apparent that human life goes through different stages (e.g., childhood, working life, raising a family, retirement). Implicit motives could be understood as being functional adaptations to the challenges of specific stages in life. For example, the entrance into college or working life might be supported by a high implicit achievement motive, whereas the implicit achievement motive might become less important in retirement (Veroff et al., 1960, 1980, 1984; Salili, 1996; McClelland et al., 1998). In addition, critical life events and specific life circumstances might also influence the implicit motive scores (Veroff et al., 1960; Salili, 1996). For example, Veroff et al. (1960) provided evidence that the loss of a spouse is related to a decrease in implicit motive scores. They argued that such powerful life events might lower the general motivational concern for most individuals and, subsequently, also their implicit motive scores.

Other researchers argued that changes in the individual perception of aging and the future time perspective (Valero et al., 2014; Thielgen et al., 2015), might cause changes in implicit motives as individuals age. Valero et al. (2014) assumed that the future time perspective decreases with advancing age, resulting in an increased urge to satisfy implicit motives and, consequently, in higher implicit motive scores in aging adults. For example, Thielgen and his colleagues concluded “that a decreasing future time perspective increases perceived importance of social relations as well as wellbeing through nurturing social contacts” (Thielgen et al., 2015, p. 196). Thus, these researchers assumed that such activities become increasingly rewarding and, consequently, activate the respective implicit motives (Valero et al., 2014).

Denzinger et al. (2016) took a different theoretical view: they argued that implicit motive scores are strongly related to affective and neuro-endocrinological systems. The authors assumed that individuals undergo some changes in their regulation of affects (e.g., older adults react less strongly to affective incentives than younger adults because they probably strive for emotional stability) and in their hormonal balance (e.g., menopause) with increasing age. They assumed that these changes influence the arousal of implicit motives and are responsible for a general decrease in implicit motive scores.

To summarize, implicit motives seem to be subject to age shifts. Researchers assumed that critical life events, role transitions, changed perceptions of aging and the future time perspective as well as maturation influence the individual implicit motive disposition. Of course, cohort effects might also have influenced the results of the above-mentioned studies. However, this would also be evidence of the susceptibility of implicit motives to historical or social-structural circumstances.

### Longitudinal analyses

Studies in this section repeatedly analyzed implicit motive scores over a period of several days (e.g., Winter, 1991) or even decades (e.g., Skolnick, 1966). Such longitudinal studies permit not only the methodologically sound investigation of inter-individual differences in stability and change but also the investigation of intra-individual stability and change. Table 3 gives an overview of research that measured implicit motive scores longitudinally. These studies generally differ in their research questions because some authors were interested in issues related to the theory of measurement (e.g., stability coefficients of implicit motive scores), whereas others were interested in factors that influence implicit motives over time. Thus, we focus in the first part on studies that were primarily interested in the retest correlation of implicit motives. In the second part, we give an overview of studies that explicitly investigated the change of implicit motives over time depending on specific influencing factors.

**Table 3:** Longitudinal studies investigating implicit motive scores.

**Author(s)**	**Sample**	**Measurements**	**Interval**	**Motive(s)**	**Results**	**Assumptions**	**Comments**
Birney, 1959	*N* = 46 (first), *N* = 40 (second), *N* = 24 (third) male college students	3	4 months, 6 months (October to April)	Achievement	Retest-correlation: 0.29 (4 months), 0.56 (6 months)	Situational influences on motive scores (academic and social concern in different months)	Reliability study
Busch and Hofer, 2012	*N* = 129 German adults, *N* = 122 Cameroonian adults	2	18 months	Power, Affiliation, Achievement	Retest-correlation Cameroonian adults: 0.22 (power), 0.15 (achievement), 0.19 (affiliation). German adults: 0.16 (power), 0.25 (achievement), 0.34 (affiliation)	Individuals stably interpret the picture cues as opportunities for motive expression	Reliability study
deCharms, 1976a	*N* = 27–61	3	1 year	Achievement	Retest-correlation: 0.14 to 0.46	No assumptions about change without training	Reliability study of control group
Franz, 1994	*N* = 48	2	10 years	Power, Affiliation, Achievement	Stability and change: decrease in *n* Ach, increase in *n* Aff, decrease in *n* Pow in men	Reduced self-concern and greater concern with communion in the transition to midlife	
Haber and Alpert, 1958	*N* = 26 male college students	2	3 weeks	Achievement	Retest-correlation: 0.54 (corrected: 0.70)	Decrease in implicit motives is due to an adaption to the testing procedure	Reliability study, study for a refinement of the TAT material, retest after arousal
Heckhausen, 1963	*N* = 42 working students	2	5 weeks	Achievement	Retest-correlation: 0.42 to 0.59	Motives are stable, but differences are due to situational influences on the measurement	Reliability study
Jenkins, 1987	*N* = 62 college-educated women	2	14 years	Achievement	Increase of *n* Ach for women in highly achievement-arousing jobs over 14 years	Accumulated occupational experiences in a more or less motive-stimulating environment	
Jenkins, 1994.	*N* = 62 college-educated women	2	14 years	Power	Increase in power (in highly power-arousing jobs e.g., teachers or psychotherapists), decrease in low power-arousing jobs.	Accumulated occupational experiences in a more or less motive-stimulating environment	
Kagan, 1959; Kagan and Moss, 1959	*N* = 86 children	3	2.5–3 years between sessions	Achievement	Retest (Phi): 0.32 (3 years later), 0.22 (6 years later), increase of achievement themes with age, but also stability over time	Tendency to seek achievement goals and influence of the parents	
Kraiger et al., 1984	*N* = 74 college students	2	1 week	Power	Retest-correlation: 0.38 to 0.52	No assumptions about change of motives	Reliability study, replication of Winter and Stewart (1977)
Lundy, 1985	*N* = 42 college students	2	1 year	Intimacy, Affiliation	Retest-correlation: 0.56 (intimacy); 0.48 (affiliation)	No assumptions about change of motives	Reliability study
Morgan, 1953	*N* = 39–62 male high school students	2	5 weeks	Achievement	Retest-correlation: 0.56 to 0.64	No assumptions about change of motives	Reliability study
Roch et al., 2017	*N* = 74 students	2	9 weeks	Power, Affiliation, Achievement	Retest-correlation: 0.37 (power), 0.36 (achievement), 0.44 (affiliation)	Implicit motives have moderate to high retest stability	Reliability study with treatment between measures
Schultheiss et al., 2003	*N* = 54 (18 normal cycling women, 18 women using oral contraceptives, 18 men)	3	Around 10 days between sessions	Power, Affiliation	Cycle dependent fluctuation of motive scores	Cycle dependent hormonal levels increase or decrease the urge to satisfy specific needs	
Schultheiss et al., 2008	*N* = 90	2	2 weeks	Power, Affiliation, Achievement	Retest-correlation: 0.39 (power), 0.61 (affiliation), 0.37 (achievement)	Participants show substantial stability from one testing occasion to the next	Reliability study
Skolnick, 1966	*N* = 93 adolescents	2	20 years	Power, Affiliation, Achievement	Men: “stability” in *n* Pow (0.34), but not *n* Ach (0.03) and *n* Aff (−0.11); Women: “stability” in *n* Ach (0.24) and *n* Aff (0.21), but not *n* Pow (0.20)	Different roles of women and men are responsible for motive differences	
Winter and Stewart, 1977.	*N* = 70 college students	2	6–8 days	Power	Retest-correlation: 0.61 (same stories are allowed), 0.27 (different stories)	Stability similar to personality traits, small retest-reliabilities are due to test instructions	Reliability study
Winter, 1973	*N* = 56 (first), *N* = 9 (second)	3	3–18 months	Power	Retest-correlation: 0.29 to 0.56	TAT responses are affected by situational and contextual cues, but also by learning and change of motives	Reliability study
Winter, 1973	*N* = 110	4	1 year, in total 4 years	Power	Retest-correlation: 0.17 to 0.30	TAT responses are affected by situational and contextual cues, but also by learning and change of motives	Reliability study
Winter, 1991	*N* = 70 college students	2	6–8 days	Power, Affiliation, Achievement	retest-correlation: −0.18 (different stories) to 0.71 (same stories are allowed);	No assumptions about change of motives	Reliability study

*n Ach, implicit achievement motive; n Pow, implicit power motive; n Aff, implicit affiliation motive*.

#### Studies that investigated retest correlations

Many of the studies in Table 3 were primarily interested in methodological questions about reliability issues when measuring the implicit motive. These studies take an approach based on classical Test Theory. Their objective is to test the quality of the implicit motive measure. Thus, the general underlying assumption of researchers conducting such studies is a high stability of implicit motives. Consequently, the respective authors rarely discussed factors that might influence implicit motives. Actually, these studies indicate reasonable retest-correlations and, consequently, a certain stability of implicit motives. Schultheiss and Pang (2007) conducted a meta-analysis including several studies on the retest-correlation of implicit motives that are also mentioned in Table 3 and briefly summarized the results in the chapter of a book. They reported a mathematically projected average stability coefficient of 0.71 for a retest interval of one day, up to 0.25 for a retest interval of ten years. Obviously, the correlation coefficient decreases as the retest interval increases. These results indicate an initial high stability of implicit motive scores, but the decreasing correlation coefficient also signalizes that some kind of change occurs over time.

How do the researchers explain and interpret the change in implicit motive scores in their longitudinal studies? As mentioned above, many scholars assumed that implicit motives remain stable over time, similar to enduring personality traits (e.g., Morgan, 1953; Haber and Alpert, 1958; Heckhausen, 1963; Winter and Stewart, 1977; Schultheiss et al., 2008). These authors attributed low stability coefficients to specific measurement problems. They concluded that differences in implicit motive scores over time are due to the use of different test forms and picture cues (Morgan, 1953; Winter et al., 1977; Lundy, 1985), to an adaption to the testing procedure by the participants (Haber and Alpert, 1958), to an implicit demand for novel stories in the second testing (Winter and Stewart, 1977), or to situational influences on the measurement (e.g., authors reasoned that not all situations are equally good opportunities for motive satisfaction; Birney, 1959; Heckhausen, 1963; Schultheiss et al., 2008). In their meta-analysis Schultheiss and Pang (2007) also assumed that different testing situations (e.g., instructions that allow the same story to be written again) and testing materials (e.g., different picture cues at the assessments) as well as stressful or motive-arousing life events might reduce the retest stability. Unfortunately, the authors of this meta-analysis went not into much detail and failed to discuss the factors that might be responsible for the stability of implicit motives. Birney (1959) argued that implicit motives (e.g., the implicit achievement motive) are highly situational in character and might be influenced over time by a change in academic and social concern. Individuals might be exposed to a high volatility of motive-arousing situations (e.g., the achievement motive of students will be aroused more during exam time than during holidays) which might lead to low retest stability coefficients (Birney, 1959). However, Winter (1973) suggested that the lower stability coefficient for implicit motive scores is not only caused by different situational and contextual cues influencing the measurement of implicit motives, but also by a real change in the implicit motives. He assumed that implicit motives underlie specific learning processes over the course of life.

#### Studies that have investigated longitudinal influences on implicit motives

Only a few studies have been directly concerned with the investigation of implicit motives' temporal dynamics using a longitudinal approach. Jenkins (1987, 1994) investigated the influence of specific employment situations on the implicit motive disposition over time. Specifically, she was interested in the changes in the implicit achievement and power motives in women over 14 years of age depending on their employment in specific occupational positions that might be more or less motive arousing. She found that occupations in jobs that stimulate specific implicit motives through their tasks and responsibilities (e.g., professors or teachers) are responsible for an increase in these implicit motives 14 years later. Other authors have also found significant increases in the implicit achievement motive over ten years subject to an achievement stimulating environment (e.g., high maternal concern with children's achievement and high educational level of the parents; Kagan, 1959; Kagan and Moss, 1959). The results of these studies indicate that implicit motives can be learned and strengthened through the steady confrontation with motive-specific incentives in adulthood, causing a reinforcement of the individual implicit motive disposition.

Franz (1994) studied the changes in implicit motives with a retest interval of ten years and documented a significant increase in the implicit affiliation motive and a decrease in the implicit achievement motive for both women and men, but a decrease in the implicit power motive only for men. However, she also reported some kind of differential stability in implicit motives: Individuals maintained their relative placement within the group in terms of their implicit achievement and intimacy motive scores (high rank-order stability). Skolnick (1966) also reported partial stability of implicit motives within a retest interval of 20 years. She found significant correlations between adolescent (17–18 years old) and adult motive scores (20 years later) in the implicit power motives of men (*r* = 0.34, *p* < 0.01), as well as a stability in women's implicit achievement (*r* = 0.24, *p* < 0.05) and, to some degree, women's affiliation motives (*r* = 0.21, *p* < 0.10).

Even a study that analyzed implicit motives scores at two or more measurement points within only a short period of time gives insights in the temporal dynamics of implicit motives. Researchers investigated the influence of hormonal fluctuations during the menstrual cycle on implicit motive scores (Schultheiss et al., 2003; Ball et al., 2014). The researchers assessed the implicit motives of women at three measurement points corresponding to the menstrual, midcycle, and premenstrual phases of women's menstrual cycle. Schultheiss et al. (2003) found cycle-dependent fluctuations in implicit affiliation and power motive scores: the implicit affiliation motive was increased and the implicit power motive was decreased at midcycle (around ovulation phase). This study documents the influence of (situational) changes in gonadal steroid hormones on implicit motives.

In the following paragraphs, we will describe the theoretical assumptions and the interpretations these authors' made in their articles about the stability of and changes in implicit motives. Skolnick (1966) argued that implicit motives are stable characteristics of one's personality, but she also assumed that they are only stable over time if they are congruent with the prevailing gender roles of the society (e.g., demand for self-assertive characteristics in men). Thus, social roles might cause changes in the individual implicit motive disposition. In addition, other researchers concluded that implicit motives change over time due to learning, age-related changes and normative life experiences (e.g., Kagan, 1959; Kagan and Moss, 1959; Winter, 1973; Jenkins, 1987, 1994; Franz, 1994). For example, Franz (1994) presented three factors that might lead to these changes: maturation or personal development; changes in normative reactions to the individual social context; and non-normative changes, such as stress and critical life events. She argued that changes in implicit motives are due to changes in the individual concern for communion and agency. Individuals in midlife experience a movement toward greater concern for other people and communion, and reduced self-concern. Higher interiority (e.g., higher responsiveness to inner stimuli, reduced self-assertiveness and reduced striving for challenges; Rosen and Neugarten, 1964) and an adaption to the environment with increasing age might be related to a change in implicit motive dispositions (e.g., decreasing implicit achievement motives).

However, changes in implicit motives may be related not only to individual developmental processes but also to characteristics of the environment and related learning experiences. For example, Jenkins (1987, 1994) argued that a changed life situation (e.g., a specific motive-arousing job) is accompanied by factors such as motive gratification and enjoyment that may lead to long-term changes in underlying motive dispositions. She assumed that specific life situations (e.g., a high-powered job) offer a motive stimulating and reinforcing environment, because daily work life situations provide cues that arouse implicit motives similarly to laboratory arousal conditions. Other authors likewise argued in the context of education that reinforced environmental factors and specific demands from the environment influence the implicit motive scores. These researchers suggested that environmental factors such as achievement demands and education of the parents (not only in childhood, but also in adolescence) encourage specific behavioral tendencies (e.g., striving for achievement goals) that, subsequently, influence the implicit motive disposition (Kagan, 1959; Kagan and Moss, 1959).

To summarize, studies investigating the stability of and changes in implicit motives longitudinally indicate that changed life situations, environmental and situational factors, learning experiences, and age-related developmental processes influence implicit motives over time. However, many studies were interested in the mere stability of the measurements. These studies indicate a certain degree of stability in the implicit motive scores according to their acceptable retest-correlation coefficients. These stability coefficients are rather high for short retesting periods, but decrease with longer retesting intervals. Authors attributed situational and temporal influences on the measurement instrument of implicit motives especially to the decreased stability coefficients, but in some cases also to a change in the individual's implicit motive disposition.

### Studies investigating stability and change using alternative measures

Over the past years, some new ways of measuring implicit motives have been developed that partially use other measuring approaches than the open fantasy-based format. For example, measures such as the MMG, the OMT or the PACT use picture cues with structured or semi-structured response formats. Other developments focus on reaction times and physiological measures (e.g., Dufner et al., 2015). The authors of these new and innovative developments claim that these tests properly assess implicit motives or at least specific facets of them. However, skeptical voices are already questioning the validity of these tests as an implicit motive measure in the McClelland-Atkinson tradition (e.g., Houwer et al., 2009). Besides, the motive scores assessed using different measures (e.g., PSE, MMG, OMT) appear to be only weakly related to each other (Schüler et al., 2015). It should be noted that the present article is only concerned with the question of stability of and changes in implicit motives, not with a comparison or a critique of implicit motive measures. Such issues have been discussed elsewhere (e.g., Schultheiss, 2008; Houwer et al., 2009; Schultheiss et al., 2009; Schüler et al., 2015). Unfortunately, only few studies using alternative measures of implicit motives investigated their stability and changes in them. Besides, these studies are frequently only concerned with reliability issues. These studies are summarized in Table 4 and described briefly in the following paragraph.

**Table 4:** Studies investigating stability and change using alternative measures.

**Author(s)**	**Measure**	**Sample**	**Measurements**	**Interval**	**Motive**	**Main results**	**Assumptions**	**Comments**
Ball et al., 2014	OMT	*N* = 66 women (33 normal cycling women, 33 women using oral contraceptives)	3	Around 10 days between sessions	Power, Affiliation, Achievement	Cycle-phase related variation only in motive enactment but not in overall motive scores	Implicit motives seem to depend on physiological incitement	
Baumann and Scheffer, 2011	OMT	*N* = 27 students	2	2 years	Achievement	Retest-correlation: 0.50 (achievement flow motive)	Reasonable stability over a period of 2 years	Reliability study
Dufner et al., 2015	Physiological assessment and IAT	*N* = 191 students	2	14 months	Affiliation	Retest-correlation: 0.42 (physiological assessment); 0.45 (IAT)	Individual differences are moderately stable across time	Reliability study
Rheinberg and Krug, 2017	MMG	Various samples with different sizes	–	Various training sessions	Achievement	Reduction in fear of failure and an increase in hope of success after achievement training in the classroom	Facets of motives are trainable	This book includes various studies on motive training
Scheffer et al., 2007	OMT	*N* = 66 male army officers	2	2 weeks	Affiliation	Retest-correlation: 0.41 to 0.72 (effect moderated by intelligence)	Implicit motives are stable because they are acquired early in life and are predictive over a long time span	Reliability study
Schmalt, 1999	MMG	*N* = 140 third- and fourth-graders	3	2 weeks and 8 weeks	Achievement	Retest-correlation: 0.67 to 0.85 (2 weeks), 0.70 to 0.79 (8 weeks)	High stability across time	Reliability study

Compared with the few studies examining training effects on implicit motives measured with the TAT/PSE, the literature on motive training using the MMG as a means of assessing implicit motive scores is comparatively rich. Rheinberg and Krug (2017) summarized these studies investigating the effects of motive training in the classroom. In sum, the authors reported significant increases in the hope of success and, especially, significant decreases in the fear of failure component of the implicit achievement motive (measured using the MMG) after training (Heckhausen and Krug, 1982; Rheinberg and Krug, 2017). Rheinberg and Krug (2017) used an explanation similar to McClelland and Winter (1969) and deCharms (1976a). They assumed that the achievement motive is a self-evaluating system that is trainable. The achievement-oriented behavior of individuals (i.e., goal setting, attribution of success and failure; success and failure related affect) seems to be driven by their hope of success and fear of failure. A change in this self-evaluation processes might change the underlying motivation and, consequently, the achievement motive (see for an overview also Heckhausen and Krug, 1982).

Another group of authors used a similar study design to that of Schultheiss et al. (2003) to examine the dynamic modulation of implicit motives during women's menstrual cycle. Ball et al. (2014) used repeated measures to investigate whether the implicit motive expression, assessed with the help of the OMT, is affected by cycle-phase-dependent fluctuations in sex hormones. They reported a cycle-phase-related variation only in implicit motive enactment (captured as a subscale of the OMT) but not in overall motive scores. In detail, they found a decreased incentive-based inhibition of the implicit power motive at the ovulation phase. Even though the authors found no evidence for cycle-phase-dependent changes in overall motive scores, this study documents the influence of gonadal steroid hormones on motive-related processes.

As already mentioned, most of the studies using these alternative measures to assess implicit motives are primarily concerned with reliability issues and investigating the retest-correlation of their measure. The authors assumed a reasonable stability of their implicit motive scores over time and report retest-correlation coefficients between 0.41 and 0.85 for measures with the OMT, MMG, PAC-AFF (a physiological measure) and the IAT with intervals of 2 weeks to 24 months (Schmalt, 1999; Scheffer et al., 2007; Baumann and Scheffer, 2011; Dufner et al., 2015). In sum, motive scores assessed using other measures show comparable retest-correlation coefficients to implicit motive scores assessed using the PSE/TAT.

## Discussion

The purpose of this article was to give an overview of studies that analyze the stability of and changes in implicit motives and to summarize the underlying theoretical assumptions of the respective authors. We first focused on studies that investigated the trainability of implicit motives. In a second step, we gave an overview of studies that use cross-sectional and longitudinal approaches to investigate the stability of and the changes in implicit motives over the course of a person's life. We will summarize and discuss the main results of these studies in the following paragraphs.

### Studies that investigated the trainability of implicit motives

Researchers who investigated the trainability of implicit motives assumed that training and exercises can strengthen the implicit motive disposition (e.g., McClelland and Winter, 1969). They supposed long-term changes and provided evidence for positive behavioral consequences even after several years. Indeed, the training programs utilized the situational susceptibility of implicit motives but, presumably, focused too little on affective (learning) experiences as the basis of changes in implicit motives. Apparently, affective experiences are of utmost importance for the arousal and the development of these associative networks (Schultheiss, 2008). Consequently, it might be difficult to strengthen them without accompanying affective experiences. This may be one reason why the long-term behavioral changes of training programs might primarily be due to other factors influenced by the training, such as improved life management skills (McClelland, 1972). Other authors even supposed that some achievement motive training programs do not change the level of the individual implicit achievement motive, but rather its direction from less fear of failure to more hope of success (for an extensive discussion on motivational trainings, see Heckhausen and Krug, 1982; Rheinberg and Engeser, 2010).

### Cross-sectional analyses

Researchers who have analyzed age differences in implicit motive scores predominantly concluded that changed life situations and transitions (e.g., retirement; Veroff et al., 1960, 1980, 1984; Salili, 1996; McClelland et al., 1998), personal views on aging (e.g., Valero et al., 2014; Thielgen et al., 2015), and maturation (e.g., Denzinger et al., 2016) might be responsible for some kind of change in implicit motives. Of course, it cannot be ruled out that generational effects are also responsible for different strengths of implicit motives in aging adults. Veroff et al. (1960) stated that these cohort effects are a possible reason for changes in implicit motive scores, though only an additional determinant to those mentioned above.

Why did some studies find significant relationships between age and implicit motives while others did not? Why did some studies only find age differences in specific implicit motives (e.g., in the implicit affiliation motive, but not in the implicit achievement motive or even only in the achievement motive)? To the detriment of research, the motivational system is a dynamic system. Implicit motives do not appear to be homogeneous across genders (e.g., Denzinger et al., 2016; Drescher and Schultheiss, 2016), different age-groups, or birth cohorts (e.g., Veroff et al., 1984). Their “stability and age-shifts are conditioned by social structural and historical circumstances” (Veroff et al., 1984, p. 1157). However, the extent and the direction of changes “may depend on the dynamics of each motive” (Veroff et al., 1984, p. 1157). Thus, the results of the studies dependent strongly on the sample and even on the year of measurement.

### Longitudinal analyses

Some researchers who have repeatedly analyzed implicit motives using a longitudinal approach have concluded that implicit motive scores change according to situational differences in the measurement of implicit motives (e.g., Morgan, 1953; Haber and Alpert, 1958; Winter and Stewart, 1977). Of course, this might be one reason for changes in implicit motive scores. However, researchers also assumed that implicit motives change through learning experiences (e.g., working in powerful positions), age-related changes (e.g., maturation and changes in the hormonal system), and specific life events (e.g., loss of a significant other; Kagan, 1959; Kagan and Moss, 1959; Winter, 1973; Jenkins, 1987, 1994; Franz, 1994).

Some studies only made assumptions about the retest correlation of the measure but not specifically about factors influencing implicit motives over time (e.g., deCharms, 1976a; Kraiger et al., 1984; Lundy, 1985; Winter, 1991). This article is not directly interested in a statistical comparison of reliability coefficients^1^, but rather in the inferences from the stability of these scores to the stability of implicit motives. High retest correlations might indicate that implicit motives remain stable over time, whereas low coefficients may imply that implicit motives change over time. The above-mentioned studies and the short meta-analysis of Schultheiss and Pang (2007) showed that the retest correlation of implicit motive scores decreases over longer retest intervals. However, the above-mentioned overall retest correlations should also be interpreted with caution because they are only projections based on relatively few studies with observed retest correlations that are mostly not very high by conventional standards. Besides, a reliability estimation over longer time periods might be problematic because low retest correlations are due to both low reliability and differential change. Unfortunately, it is not possible to disentangle the relative contributions of error and change. Thus, low retest-correlations do not necessarily imply differential change in the construct. However, comparable retest correlations have been reported for personality traits that were assessed via questionnaire, too (e.g., Schuerger et al., 1989). Thus, one might assume that the stability of and changes in implicit motives are very similar to the stability of and change in other personality characteristics such as characteristic adaptions (e.g., values, McAdams and Pals, 2006).

### Stability or change over the course of life?

Individual differences in implicit motives are assumed to develop through affective learning experiences (McClelland et al., 1989). The assumption that these learning processes primarily take place during early, preverbal childhood seems to be untenable. Some researchers on this topic even assumed that implicit motives can “be formed at any time in life” (McClelland, 1958, p. 452) and that their strength can change during the course of life as a result of emotional learning (Schultheiss and Brunstein, 1999). Individuals gain many different experiences throughout their lives which are affectively rewarding, too. There are always new situations that arouse implicit motives (e.g., a new job in a high-achievement environment). Therefore, it is hardly surprising that affective experiences in life influence the strength of implicit motives.

The objection may be raised that childhood in particular is characterized by strong learning experiences and that these affective learning processes decrease in adulthood (for a similar argument see McClelland, 1958). It is conceivable that implicit motives might develop faster in childhood than in adulthood because adults frequently have contradictory affective experiences (e.g., a striving for influence does not lead to well-being but to negative consequences) which cannot easily be unlearned and forgotten and, thus, might complicate the change of implicit motives. Nevertheless, even characteristics that are obviously developed in childhood and that are assumed to be very stable, such as the attachment style, only display moderate stability coefficients (e.g., Fraley, 2002) and also change as a result of specific life events (e.g., Hamilton, 2000; Waters et al., 2000). Thus, there are indeed situations in life that even influence human attributes that are presumed to be very stable.

Furthermore, implicit motives are related to many other factors, such as hormones (e.g., Schultheiss, 2013). It is possible that such elements have an impact on implicit motives because these influencing parameters depend on situational (e.g., stress) and age-related changes (e.g., menopause).

Another issue is that the implicit motive measure actually displays both trait-stability and state-variability (cf., Fleeson, 2001). A person might have a stable implicit motive disposition, similar to a trait. However, the actual strength of the related motivation could also vary over time and with respect to available arousing cues. A clear example of this phenomenon is appetite. For example, the attractiveness of available food and the number of hours since the last meal affect appetite and the eating behavior. It cannot be ruled out that changes in implicit motive scores are merely due to this effect. Thus, it is necessary to analyze the temporal dynamics of implicit motives considering studies that measure implicit motives across different age groups and over time in order to draw more detailed conclusions. However, the increased arousal heightens the salience of the respective associative network of the implicit motives (e.g., McClelland and Winter, 1969) and the rewarding character of specific cues. If changes in the motivational state are accompanied by corresponding affective consequences, this might lead to affective learning effects that influence the implicit motive disposition in the long run. Thus, we assume that these short-term influences and state changes might be a necessary precondition for long-term changes in implicit motive disposition.

How can the findings from the existing research be summarized? At first glance, there are different and partially inconsistent results, as well as different arguments about influencing factors contributing to these results. However, on closer inspection there is a lot of common ground. Table 5 gives an overview of factors that are said to influence implicit motive scores. Obviously, not only the sensitivity of the measure is responsible for changes in implicit motive scores (e.g., situational and temporal influences on fantasy production), as often mentioned. The majority of the scholars' arguments are built upon basic theoretical ideas that assume that, because of their individual motive disposition, people are very sensitive to specific cues which allow affective satisfaction (Schultheiss, 2008). The individual experience with different cues gives rise to the emergence of an associative implicit motive network (McClelland and Winter, 1969). The confrontation with relevant cues (physiologically and psychologically) activates and arouses this network. Access to this network might be facilitated and its strength increased if the individual is repeatedly confronted with such cues in the long-term. Besides, a changed life situation (e.g., through the transition to retirement) might have an effect on the rewarding character of specific cues. Consequently, this might change the implicit motive disposition in the long term. Even possible cohort effects are an indicator of this dynamic system. In sum, individuals adapt to the demands of life.

**Table 5:** Factors that might influence implicit motive scores.

**Factor**	**Example**
Sensitivity of the measurement instrument	Different picture cues at second measuring point, increased fantasy production
Physiological arousal of the motivational system	Sympathetic activation through the release of hormones
Psychological arousal of the motivational system	Anticipation of affective rewards
Reduced motivational concern	Loss of a spouse
Maturation, individual developmental processes	Hormonal changes due to menopause
Changed living conditions	Transition to retirement, job experiences

*This is a summary of the theoretical assumptions of the particular researchers mentioned in the results section of this article*.

However, there are a lot of unanswered questions for future research. For example, it is still unclear exactly how long implicit motives take to change. Is it possible to change the implicit motive disposition through a single critical life event, or does it take several years or decades? How strong does the affective learning experience need to be? Does it depend on the specific predispositions of the individual, such as a high openness for experience or high self-consciousness, whether implicit motives are susceptible to change? How can knowledge about lifetime changes in implicit motives be used for practical purposes, such as training programs, psychoeducation, or therapy? In sum, there is a lot of work to be done.

### What can be learned from this review?

This review gives an informed overview of studies and theoretical assumptions about the stability of and changes in implicit motives. It is a comprehensive inventory of research on this topic. Scholars have investigated numerous research questions in this field but, unfortunately, have not always been directly concerned with the susceptibility and the temporal dynamics of implicit motives. Thus, the authors of the respective studies have chosen different research questions (e.g., Is the retest reliability of the implicit motive measure sufficient? Does the career choice influence the implicit motive disposition?) but also different methodological approaches. Without doubt, some of these approaches are accompanied by widely varying methodological problems. For example, the results of cross-sectional studies might also be explained by specific cohort effects. Furthermore, some studies have investigated very restricted samples or age groups. A few older studies might have limited statistical power due to their very small sample sizes. This gives rise to numerous problems that are currently being addressed in the debate of reproducibility of psychological research (e.g., Maxwell et al., 2008; Open Science Collaboration, 2015; Smaldino and McElreath, 2016). For example, this might be related to an increased false-discovery rate (Ioannidis, 2005) and inflated effect-size estimates (Maxwell et al., 2015). Thus, a lack of statistical power is a huge problem for the explanatory power and the comparison of the results. Even several of the longitudinal studies were done within a small time period, which limits their results. Unfortunately, our database is very narrow, making the statistical comparison of the results very difficult if not impossible. Thus, this article focusses exclusively on a narrative overview of studies, their results, and, especially, on the arguments of their authors about the stability of and changes in implicit motives. We have addressed studies with different research questions and methodological approaches to increase the generalizability of the assumptions about implicit motive stability and change. Future research on this topic should avoid the above-mentioned problems and, for example, employ a quasi-experimental longitudinal design, following children from birth through different stages of life. Studies should analyze individual life trajectories and definitely take possible life circumstances and different contexts into account while, at the same time, collecting data on affective and physiological correlates. Moreover, it might be helpful to score implicit motives in people's diaries or in their literary productions. Large sample sizes are necessary because this supports the investigation of even small changes in the implicit motive disposition with high statistical power. There is considerable evidence of mean-level and rank-order changes in implicit motives. Future research should therefore address questions regarding the conditions for, the amount and the direction of change. In conclusion, the final statement of this article refers back to the initial quote of Benjamin Franklin: “When you're finished changing, you're finished.” The reviewed research suggests that implicit motives adapt to life circumstances.

## Author contributions

Both authors designed and discussed the structure of this review. The first author was responsible for the literature search and for summarizing the results. He also wrote the first draft of this paper. The second author refined and sharpened the draft of this review.

### Conflict of interest statement

The authors declare that the research was conducted in the absence of any commercial or financial relationships that could be construed as a potential conflict of interest.

## Footnotes

^1^Readers interested in methodological issues should also turn their attention to articles and reviews that address the reliability of implicit motive measurements (e.g., Entwisle, 1972; Schultheiss and Pang, 2007), discussions about these issues (e.g., McClelland, 1985; Smith, 1992b), as well as determinants (e.g., incentives, priming) of the content of fantasy stories (e.g., Klinger, 1971).
